# Psychometric Evaluation of Screens for Common Mental Disorders, Severe Mental Disorders, Substance Use Disorders, and Suicide Risk in Mozambican Healthcare

**DOI:** 10.18103/mra.v12i8.5294

**Published:** 2024-08-31

**Authors:** Kathryn L Lovero, Melissa A Stockton, M Claire Greene, Cale Basaraba, Saida Khan, Dirceu Mabunda, Flavio Mandlate, Lidia Gouveia, Wilza Fumo, Melanie M Wall, Cristiane S Duarte, Maria A Oquendo, Milton L Wainberg

**Affiliations:** 1 Department of Sociomedical Sciences, Columbia University Mailman School of Public Health, New York, USA; 2 Department of Psychiatry, University of Pennsylvania Perelman School of Medicine, Philadelphia, USA; 3 Program on Forced Migration and Health, Heilbrunn Department of Population and Family Health, Columbia University Mailman School of Public Health, New York, USA; 4 Department of Population Health, Grossman School of Medicine, New York University; 5 Department of Mental Health, Ministry of Health, Maputo, Mozambique; 6 Department of Psychiatry, New York State Psychiatric Institute and Columbia University Vagelos College of Physicians and Surgeons, New York, USA; 7 Department of Biostatistics, Columbia University Mailman School of Public Health, New York, USA

## Abstract

Globally, mental and substance use disorders are a leading cause of disease burden. In low- and middle-income countries, where there is an extreme shortage of trained mental health specialists, validated, brief screening tools for mental and substance use disorders are required for non-specialists to efficiently identify patients in need of mental health care. Mozambique, one of the poorest countries in the world, has fewer than two mental health specialists for every 100,000 people. In the present study, we evaluated a comprehensive set of seven measures for depression, anxiety, somatization, alcohol use disorder, substance use disorder, psychosis and mania, and suicide risk among N=911 Mozambican adults in general healthcare settings. All instruments demonstrated acceptable internal consistency (α > 0.75). Compared to diagnoses made by the Mini International Neuropsychiatric Interview, all measures showed good criterion validity (AUC > 0.75), except the Psychosis Screening Questionnaire, which showed low sensitivity (0.58) for psychotic disorder. No substantial differences were observed in internal consistency when stratifying by gender, age, education level, primary language, facility-type, and patient status; criterion validity showed some variability when stratified by sub-population, particularly for education, primary language, and whether the participant was seeking care that day. Exploratory factor analyses indicated that the measures best differentiate categories of diagnoses (common mental disorder, severe mental disorders, substance use disorders, and suicide risk) rather than individual diagnoses, suggesting the utility of a transdiagnostic approach. Our findings support the use of these measures in Mozambique to identify common mental disorders, substance use disorders, and suicide risk, but indicate further research is needed to develop an adequate screen for severe mental disorders. Given the limited mental health specialists in this and other LMIC settings, these brief measures can support non-specialist provision of mental health services and promote closure of the treatment gap.

## Introduction

Globally, mental and substance use disorders (MSUD) are a leading cause of disease burden ^1^. However, in low- and middle-income countries (LMIC), where over 80% of the world’s population resides ^2^, there is an extreme shortage of trained mental health specialists ^3^. To close the mental health treatment gap, many LMIC have focused on developing task-shifted mental health services in primary care clinics and community settings ^4^. Lengthy mental health diagnostic interviews require significant time, training, and experience to administer. Brief screening instruments provide an efficient alternative for non-specialists to identify people in need of mental health services. However, few brief screening tools for MSUD have been validated in LMICs ^5^.

In Mozambique, one of the poorest countries in the world, there are fewer than two mental health specialists for every 100,000 people, almost 50 times less than in high income countries ^6^. Owing to the lack of specialists, patients seen in Mozambican mental health services are primarily limited to those with the severe mental disorders and neurological conditions ^6–7^, such as schizophrenia and epilepsy, despite other MSUD being more prevalent in the general population ^8^. To better meet mental health needs nationwide, the Ministry of Health has begun training primary care and community health workers to deliver interventions for common mental disorders (e.g., depression and anxiety), alcohol and substance use disorders, and suicide risk as well as provide referrals for severe mental disorders (i.e., psychotic disorders, mania) that require specialist attention ^9^. While the Patient Health Questionnaire-9 (PHQ-9) and the Alcohol Use Disorder Identification Test (AUDIT) have been demonstrated to be valid for identification of depression and alcohol dependence, respectively, in a Mozambican adult population ^10,11^, validated screens for the identification of other disorders to support comprehensive identification of MSUD by non-specialists are lacking. Moreover, the ability of screening tools to differentiate among disorders in Mozambican populations has not been assessed.

In the present study, we sought to contextually adapt and assess the ability of eight different screening tools to identify and differentiate depression, anxiety, somatization, alcohol use disorder, substance use disorder, psychosis, mania, and suicide risk among Mozambican adults in general healthcare settings. We worked with a team of Mozambican mental health specialists to adapt screens and assess their comprehensibility with Mozambican adults attending health units. At two primary care facilities and one tertiary care facility in Mozambique, we administered screening tools along with a gold-standard diagnostic interview, the Mini International Neuropsychiatric Interview (MINI) Plus. Findings from this study can directly support improvements in mental health services in Mozambique and may also be informative for other LMIC, especially Lusophone countries, looking to adopt brief screening measures as a method to identify individuals with MSUD and close the global mental health treatment gap.

## Methods

### SAMPLE

We conducted a cross-sectional study at two primary care facilities and one tertiary care facility in Maputo, Mozambique ^12^. Data collection occurred from May 16^th^ to June 8^th^, 2018. Each day, research assistants provided a study overview and invitation to participate in the health unit outpatient waiting areas. In the tertiary care facility, due to its size, the study description was provided in waiting areas of specific health departments serving adults (e.g., maternal and child health, adult screening services, emergency room, chronic illnesses); in the primary care facilities there was only one waiting area for all services and the study description was provided there. In the final week of data collection (June 4^th^ – June 8^th^, 2018), inclusion was specifically targeted to patients seeking mental health services to reach minimum numbers of cases needed for measure validation. Patients awaiting various health services and their accompanying family members and friends who expressed interest in participating in the study were taken to a private area within the health facility to be assessed for eligibility. Potential participants were excluded if they were less than 18 years old and/or were unable to sufficiently communicate in Portuguese, determined by interviewers asking potential participants to repeat the objectives of the study in their own words. Those who met eligibility requirements then underwent written informed consent procedures. We aimed to enroll a minimum of 400 people with at least one psychiatric diagnosis and 400 who did not meet criteria for any psychiatric diagnosis to ensure margins of error of ±5% for sensitivity and specificity estimates. Additionally, for each of the psychiatric diagnoses we aimed to enroll at least 40 participants who met diagnostic criteria for each disorder evaluated, balanced by gender, to assess criterion validity of screening tools (Table 1). All study procedures were approved by the Ethics Councils of Eduardo Mondlane University (CIBS FM&HCM/54/2017) and the New York State Psychiatric Institute Institutional Review Board (#7479). We report how we determined our sample size, all data exclusions, all manipulations, and all measures in the study.

A total of 1033 people were screened for eligibility; seven (0.7%) were under 18 years old and eight (0.8%) were not fluent in Portuguese. Twenty-nine (2.8%) of the 1018 eligible people did not provide informed consent; 78 (7.9%) of the 989 enrolled participants who did not complete all assessments (described below) were excluded from these analyses.

### MEASURES

#### Sociodemographic information

Participants self-reported sociodemographic data including age, gender, marital status, living situation (with family, friends, or other), education, religion, preferred language, ethnic group, occupation, and monthly household income.

#### Mental and Substance Use Disorder Diagnosis

MSUD diagnoses were made using the Brazilian version of the MINI Plus ^13,14^, a structured diagnostic interview ana and widely-used reference standard ^5^. We administered modules for diagnosis of current: major depressive episode, manic episode, panic disorder, posttraumatic stress disorder (PTSD), alcohol abuse/dependence, substance abuse/dependence, psychotic disorders, generalized anxiety disorder, somatization disorder, and suicide risk.

#### Battery of Mental Health Screening Measures

We adapted and administered nine structured instruments commonly used globally to screen for specific MSUD and to assess functioning, outlined in Table 1 ^15–23^.

#### Instrument Adaptation

For all instruments, we followed the four-step WHO process of translation and adaptation to prepare them for use in Mozambican healthcare settings – 1) forward translation, 2) expert panel back-translation, 3) pre-testing and cognitive interviewing, and 4) final version ^24^. With exception for the Psychosis Screening Questionnaire (PSQ) and Primary Care Post-Traumatic Stress Disorder Screen (PC-PTSD), we began with existing Portuguese translations, from Brazil or Portugal, and local research team members (FM, DM, LG, SK and LM, all bilingual in English and Portuguese) made minor adjustments for the Mozambican context (e.g., local terms for specific substances). The PSQ and PC-PTSD were translated from English to Portuguese by a professional translator and reviewed by the local research team. All instruments were back-translated by a native English speaker fluent in Portuguese and unfamiliar with the original instruments. Backtranslations were reviewed for translation accuracy by an independent measurement specialist at Columbia University and the local research team; discrepancies were resolved through group discussion and consensus decision. Instruments were field tested using cognitive interviews in 2 primary care health centers and 3 tertiary care hospitals, including a psychiatric hospital. N=84 participants were recruited who responded to the screening tool battery and N=16 to the MINI Plus. No changes were required.

#### Administration Procedures

Data were collected in face-to-face interviews. All participants first self-reported on sociodemographic information. Participants then responded to the MINI and battery of assessment scales in a randomized order (i.e., MINI followed by the battery of assessment scales or vice-versa), determined by the randomization module in REDCap. Table 1 shows the order of administration for the instruments in the assessment battery; the AUDIT and ASSIST were order-randomized owing to both questionnaires including assessment of alcohol use.

All questionnaires and interview responses were digitally recorded by interviewers using the REDCap data collection platform, a metadata-driven methodology and workflow process for providing translational research informatics support, hosted at the Foundation for Professional Development in Pretoria ^25^.

### DATA ANALYSIS

Study analyses and reporting follow the Standards for Reporting of Diagnostic Accuracy Studies (STARD) ^26^. As psychometrics of the PC-PTSD in this sample are reported elsewhere ^27^, the PC-PTSD was not included in analyses for the present study. To assess the internal consistency of scales, Cronbach’s alpha and McDonald’s omega were calculated for each scale in the whole sample, and then in subsamples stratified by age category, gender, facility type, education, and patient status. Although Cronbach’s alpha and McDonald’s omega often lead to similar results in applied settings, McDonald’s omega is subject to fewer and more realistic assumptions and has less risk of over- or under-estimation ^28^. Accordingly, both were calculated along with bootstrapped 95% confidence intervals to follow best practices for assessing internal consistency ^29^.

To assess criterion validity of most scales (PHQ-9, GAD-7, SSS-8, AUDIT, ASSIST-Alcohol, ASSIST-Cannabis, and C-SSRS) against their corresponding MINI diagnosis (e.g. MINI Depression for PHQ-9), each scale’s sum score was computed, a receiver-operating-characteristic curve (ROC) was constructed, and the area-under-the-curve (AUC) along with its 95% confidence interval were calculated. The AUC is reported in the full sample and then in subsamples stratified by gender, age category, education, preferred language, facility type, and patient status. The detailed breakdown of the ROC curves, showing sensitivity and specificity for predicting the relevant MINI diagnosis at every cutoff in the full sample, are also reported for these scales. For the PSQ, which produces only a positive/negative screen for psychosis rather than a sum score, the sensitivity and specificity of this classification against MINI Psychosis and MINI Mania diagnoses are reported in the full sample, and then for each stratified subsample.

To assess the discriminant validity of the PHQ-9, GAD-7, SSS-8, AUDIT, ASSIST-Alcohol, PSQ, and C-SSRS scales, an exploratory factor analysis (EFA) was conducted which included all scoring items from these scales and explored models with three to seven factors. The range for the number of factors explored was based on the “natural break” in the scree-plot of eigenvalues and the number of scales included in the analysis ^30,31^. Due to very few endorsements of any items in the ASSIST-Cannabis subscale, it was not included in this analysis. We compared the fit for each number of factors through multiple indices of fit and the interpretability of the goemin-rotated factor loadings. We report the comparative fit index (CFI), Tucker-Lewis index (TLI), root mean square error of approximation (RMSEA), and standardized root mean square residual (SRMR), with CFI and TLI values greater than 0.95, RMSEA values less than 0.05, and SRMR values <0.07 indicating good fit ^32,33^.

All data cleaning, criterion validity, and internal consistency analyses were conducted using R version 4.0;^34^ the exploratory factor analysis was performed in Mplus version 8.1 ^35^.

## Results

### PARTICIPANTS

Among the 911 included participants, 570 (62.6%) were female and the mean age was 32.0 years (SD=11.3). Based on MINI diagnoses, more than half of participants (51.6%, n=470) had one or more MSUD: 33% (n=298) with major depressive episode, 4% (n=33) with panic disorder, 7% (n=65) with generalized anxiety disorder, 1% (n=13) with somatization disorder, 13% (n= 115) with alcohol abuse/dependence, 2% (n=22) with substance abuse/dependence, 26% (n=235) with psychotic disorder, 8% (n=70) with manic episode, and 9% (n=86) with moderate to high suicide risk. While 33 (3.6%) participants reported past-year cannabis use, less than 5 reported past-year use of cocaine, amphetamines, inhalants, sedatives, hallucinogens, or opioids. As such, for all analyses, only items for the ASSIST assessing alcohol use (ASSIST-Alcohol) and cannabis use (ASSIST-Cannabis) were evaluated.

### INTERNAL CONSISTENCY

Table 2 presents the internal consistency of all measures in the full sample. The ASSIST-Cannabis showed excellent internal consistency (Cronbach’s alphas and McDonald’s omega >0.90). The PHQ-9, GAD-7, AUDIT, ASSIST-Alcohol, and CSSR had good internal consistency (0.80-0.89), and the SSS-8 and PSQ had acceptable internal consistency (0.70-0.79). No substantial differences were observed in internal consistency when stratifying by gender, age, education level, primary language, facility-type, and patient status (Table S1).

### CRITERION VALIDITY

We next evaluated performance of each measure compared to corresponding MINI diagnoses (Table 3, Tables S2–S3). The GAD-7, SSS-8, AUDIT, ASSIST-Alcohol, ASSIST-Cannabis, and CSSR all demonstrated good criterion validity (AUC=0.80-0.89). The PHQ showed acceptable criterion validity for depression (AUC=0.75). Because the PSQ score is dichotomous, sensitivity and specificity of screening positive on the PSQ (score of 1) were calculated instead of an AUC. The PSQ had acceptable sensitivity for mania (0.73), but poor sensitivity for psychosis (0.58). The PSQ had acceptable specificity for both psychosis and mania diagnoses (0.79 and 0.73, respectively). Evaluating performance of scales in stratified samples (Table S4), unacceptable AUC (<0.70) were observed on the GAD-7 for participants with less than primary education; the ASSIST-Cannabis for participants with greater than secondary education; and the GAD-7, SSS-8, and ASSIST-Cannabis for participants whose primary language was not Portuguese. PSQ specificity for psychosis did not improve to an acceptable level for any strata; the PSQ sensitivity and specificity for mania was below acceptable (<0.70) for participants who were themselves attending services that day.

### DISCRIMINANT VALIDITY

Finally, we conducted an EFA to assess discriminant validity of items in the scales. ASSIST-Cannabis items were removed from the EFA owing to very few non-zero responses. Based on the screen plot and the number of scales (Figure S1), we examined performance of models with 3-7 factors (Table S5–S9). All models showed a good fit, with RMSEA < 0.05, CFI and TLI ≥ 0.95, and SRMR < 0.07 (Table 4). Across factor models, items from the PHQ-9, GAD-7, and SSS-8 consistently had strong loadings onto one single factor; items from the ASSIST-Alcohol and AUDIT repeatedly loaded onto a second factor; and items from the CSSR onto a third. In the 3-factor model, items from the PSQ loaded most strongly on the factor with items from the PHQ-9, GAD-7, and SSS-8. In models with four or more factors, PSQ items consistently loaded together onto a fourth factor.

## Discussion

In this cross-sectional validation study, we compared the performance of 8 different screening instruments against the MINI Plus diagnostic gold standard in Mozambique, a country with very limited mental health specialists where, like other LMIC, brief valid screening tools for mental and substance use disorders are required for non-specialists to efficiently identify patients in need of mental health care. All instruments underwent a robust process of translation and adaptation. All instruments demonstrated good internal consistency and, except for the PSQ, criterion validity. There was some variability in the performance of the GAD-7, SSS-8, ASSIST-Cannabis, and PSQ when stratified by sub-population, particularly for education, primary language, and whether the participant was seeking care that day. We also assessed discriminant validity using EFA including all measures except the ASSIST-Cannabis.

All tools demonstrated at least good internal consistency, with Cronbach’s alphas or McDonald’s omegas above 0.78 and with no significant differences evident by sub-population. Further, all instruments that produced a sum score yielded good criterion validity, with AUC values above 0.75. In Mozambique, two recent studies have validated PHQ-9 and AUDIT, both using the MINI-5 as the diagnostic gold standard ^10,11^. In these studies, the PHQ-9 demonstrated good internal consistency and criterion validity, with a Cronbach’s alpha of 0.84 and AUC of 0.81(95% CI: 0.73, 0.89) ^10^ and the AUDIT yielded acceptable internal consistency (Cronbach’s alpha of 0.74) and good criterion validity AUC 0.94 (95% CI: 0.91, 0.96) ^11^. Other studies examining the PHQ-9 ^36–46^, GAD-7 ^47^, AUDIT ^48–54^, and ASSIST^55^ in the region have yielded similar findings. However, the SSS-8 and the PSQ – the only tool which does not produce a sum score, but a positive/negative screen for psychosis and mania – only demonstrated acceptable internal consistency. As neither the SSS-8 nor the PSQ have been validated in the sub-Saharan region, further investigation is needed to ensure valid tools are available to screen for somatization, psychosis and mania in this setting.

There were differences in the performance of some measures when stratified by various sub-populations. The PSQ failed to demonstrate acceptable (≥0.70) sensitivity or specificity among participants who were patients seeking or receiving care the day they were screened. This may be because we recruited a healthcare facility-based sample and patients’ physical illness may have influenced their mental status ^56^. The GAD-7, SSS-8, ASSIST-Cannabis performed less well among participants whose primary language was not Portuguese. Given that not all patients in the target population speak primarily Portuguese, this suggests the screening tools may need to be translated into local dialects to be better understood by patients and ultimately improve the performance of the measures. Further, ensuring local translation of mental health diagnostic and symptom terminology may improve the cultural-appropriateness and delivery of mental health screening and treatment services ^57^. Additionally, the GAD-7 performed less well among those with less education, while the ASSIST-Cannabis performed less well among those with more education, though the number of cases among this group was very low and estimates therefore less stable. Of the prior studies that assessed the validity of these measures in SSA, neither looked at differences by educational attainment ^47,55^. Future validation studies in the region should investigate these tools performance by sub-group, to ensure that they perform well across populations.

Results of the discriminant validity analyses revealed that screening tools items differentiated common mental disorders, alcohol and substance use disorders, thought disorders, and suicide risk. There was limited discrimination among the three tools for common mental disorders, including major depressive, anxiety, and somatization disorders. Rather than a flaw in the tools, these findings are likely reflective of shared symptomology and comorbidity of these disorders. The factors identified in our EFA are consistent with empirically supported clustering of disorders as described in the DSM-5 ^58^. Similarly, the factors found here align with recent research demonstrating the same latent transdiagnostic factors across specific disorders ^5,59,60^. These findings support recommendations for using transdiagnostic assessment and intervention approaches ^61,62^.

In the present study, we employed multistep adaptation process in collaboration with local mental health specialists to adapt and validate a battery of brief screening tools for comprehensive assessment of MSUD, the first to do so in Mozambique and one of the first in the sub-Saharan region. Despite these strengths, our study should be considered in light of the following limitations. For one, we used a health facility-based sample and thus our findings may not be generalizable to the general population. Further, we used a targeted enrollment strategy for part of the inclusion period to ensure we would recruit an adequate number of participants with each disorder assessed. Thus, we did not calculate the positive predictive value or the negative predictive value of the tools.

## Conclusions

Few brief screening measures for MSUD have been adapted for use in LMIC. Our data support the validity of seven measures for assessing depression, anxiety, somatization, alcohol use disorder, substance use disorder, psychosis, and mania in Mozambican adults. Given the limited mental health specialists in this and other LMIC settings, these brief measures can support non-specialist provision of mental health services and promote closure of the treatment gap. Despite the breadth of disorders adequately captured by the measures assessed in this study, future research is needed to identify an adequate brief measure for identification of psychosis. Additionally, as the present study was conducted in healthcare settings, future work should determine if measures evaluated here perform similarly in community-based samples.

## Supplementary Material

Supplementary material

## Figures and Tables

**Table 1.** Battery of mental health screening measures.

Order	Measure	Corresponding MINI Diagnoses
1	WHO Disability Assessment Schedule 2.0	N/A
2	Patient Health Questionnaire 9	Major Depressive Episode
3	Generalized Anxiety Questionnaire 7	Generalized Anxiety Disorder, Panic Disorder
4	Primary Care PTSD Screen for DSM- 5	PTSD^b^
5	Somatic Symptom Scale	Somatization Disorder
6/7^a^	Alcohol Use Disorder Identification Test	Alcohol Abuse/Dependence
6/7^a^	Alcohol, Smoking & Substance Involvement Screening Test	Substance Abuse/Dependence
8	Psychosis Screening Questionnaire	Psychotic Disorder, Manic Episode
9	Columbia Suicide Severity Rating Scale	Suicidality

*Note*. N/A = not applicable; MINI = Mini International Neuropsychiatric Interview; WHO = World Health Organization; PTSD = post-traumatic stress disorder; DSM-5 = Diagnostic and Statistical Manual of Mental Disorders, 5th Edition

a The order of administration of these measures was randomized.

b Data reported in Massinga LJ et al.’s “Screening for post-traumatic stress disorder (PTSD) in Mozambique: Validation of the Primary Care-PTSD Screen for DSM-5 (PC-PTSD-5)”.^27^

**Table 2.** Internal consistency of each measure in overall sample.

	Cronbach’s α (95% CI)	McDonald’s ω_t_ (95% CI)
PHQ-9	0.828 (0.804, 0.849)	0.828 (0.805, 0.849)
GAD-7	0.830 (0.804, 0.852)	0.831 (0.805, 0.853)
SSS-8	0.792 (0.759, 0.821)	0.794 (0.762, 0.823)
AUDIT	0.894 (0.877, 0.907)	0.895 (0.879, 0.909)
ASSIST-Alcohol	0.869 (0.847, 0.892)	0.869 (0.849, 0.893)
ASSIST-Cannabis	0.910 (0.868, 0.936)	0.913 (0.875, 0.938)
PSQ	0.784 (0.776, 0.824)	0.794 (0.788, 0.831)
CSSR	0.845 (0.800, 0.875)	0.849 (0.809, 0.879)

Note. PHQ-9 = Patient Health Questionnaire 9; GAD-7 = Generalized Anxiety Questionnaire 7; SSS-8 = Somatic Symptom Scale 8; AUDIT = Alcohol Use Disorder Identification Test; ASSIST = Alcohol, Smoking & Substance Involvement Screening Test; PSQ = Psychosis Screening Questionnaire; CSSR = Columbia Suicide Severity Rating Scale.

**Table 3.** Criterion validity of each measure in overall sample.

	Comparator MINI Diagnosis	Proportion w/MINI Diagnosis (N)	AUC (95% CI)
PHQ-9	Depression	0.33 (298)	0.754 (0.720, 0.787)
GAD-7	Panic	0.04 (33)	0.828 (0.762, 0.895)
GAD-7	Generalized Anxiety	0.07 (65)	0.818 (0.746, 0.889)
SSS-8	Somatization	0.01 (13)	0.845 (0.747, 0.943)
AUDIT	Alcohol	0.13 (115)	0.883 (0.846, 0.919)
ASSIST-Alcohol	Alcohol	0.13 (115)	0.863 (0.823, 0.903)
ASSIST-Cannabis	Substance	0.02 (22)	0.834 (0.734, 0.934)
PSQ	Psychosis	0.26 (235)	Sens.: 0.579 (0.514, 0.638) Spec.: 0.791 (0.760, 0.821)^a^
PSQ	Mania	0.08 (70)	Sens.: 0.729 (0.629, 0.829) Spec.: 0.731 (0.702, 0.761)^a^
CSSR	Mod-High Risk	0.09 (86)	0.895 (0.856, 0.933)

*Note*. PHQ-9 = Patient Health Questionnaire 9; GAD-7 = Generalized Anxiety Questionnaire 7; SSS-8 = Somatic Symptom Scale 8; AUDIT = Alcohol Use Disorder Identification Test; ASSIST = Alcohol, Smoking & Substance Involvement Screening Test; PSQ = Psychosis Screening Questionnaire; CSSR = Columbia Suicide Severity Rating Scale; Sens. = sensitivity; Spec. = specificity.

a No AUC for PSQ because 0 and 1 are only possible scores.

**Table 4.** Exploratory factor analysis of measures considering 3-, 4-, 5-, 6-, and 7-factor models.

# Factors	Model χ^2^	CFI	TLI	RMSEA (90% CI)	SRMR
3	2,428	0.95	0.95	0.034 (0.032, 0.036)	0.066
4	2,054	0.97	0.96	0.030 (0.028, 0.032)	0.060
5	1,740	0.98	0.97	0.026 (0.024, 0.028)	0.052
6	1,483	0.98	0.98	0.022 (0.019, 0.024)	0.048
7	1,325	0.99	0.98	0.020 (0.017, 0.022)	0.045

Note. CFI = comparative fit index; TLI = Tucker–Lewis index; RMSEA = root mean square error of approximation; SRMR = standardized root mean square residual.
