# Supplementary material for: Psychometric Evaluation of Screens for Common Mental Disorders, Severe Mental Disorders, Substance Use Disorders, and Suicide Risk in Mozambican Healthcare
Source: Med Res Arch. Author manuscript; Available in PMC 2024 Sep 12. (PMC11391859; doi:10.18103/mra.v12i8.5294)

Table S1. Internal consistency of scales by gender, age, education level, primary language, facility-type, and patient status.

	PHQ-9		GAD-7		SSS-8		AUDIT		ASSIST Alc		ASSIST Cann		PSQ		CSSR	
	α	ω_t	α	ω_t	α	ω_t	α	ω_t	α	ω_t	α	ω_t	α	ω_t	α	ω_t
**Overall** (N=911)	0.828	0.828	0.830	0.831	0.792	0.794	0.894	0.895	0.869	0.869	0.910	0.913	0.784	0.794	0.845	0.849
**Gender**																
Female (N=570)	0.835	0.835	0.840	0.841	0.802	0.804	0.880	0.881	0.845	0.847	0.941	0.951	0.786	0.795	0.857	0.861
Male (N=341)	0.818	0.819	0.815	0.815	0.772	0.777	0.887	0.889	0.865	0.866	0.906	0.909	0.779	0.792	0.799	0.812
**Age**																
18-25 (N=324)	0.826	0.827	0.822	0.823	0.798	0.799	0.881	0.882	0.878	0.879	0.913	0.915	0.775	0.784	0.884	0.887
> 25 (N=587)	0.828	0.829	0.835	0.836	0.792	0.796	0.899	0.90	0.864	0.865	0.908	0.914	0.789	0.799	0.794	0.803
**Education**																
< Primary (N=183)	0.797	0.799	0.751	0.754	0.763	0.769	0.912	0.914	0.885	0.887	0.938	0.943	0.764	0.775	0.888	0.891
< Secondary (N=483)	0.827	0.828	0.833	0.833	0.808	0.810	0.894	0.896	0.859	0.860	0.892	0.897	0.790	0.8	0.795	0.804
≥ Secondary (N=244)	0.853	0.855	0.859	0.860	0.778	0.782	0.867	0.869	0.868	0.869	—[a]	—	0.787	0.795	0.879	0.883
**Language**																
Portuguese only (N=517)	0.800	0.801	0.817	0.817	0.781	0.784	0.902	0.903	0.886	0.886	0.912	0.916	0.789	0.799	0.869	0.872
Portuguese + other (N=202)	0.865	0.866	0.850	0.851	0.836	0.837	0.888	0.891	0.830	0.836	0.906	0.910	0.791	0.798	0.812	0.819
Other only (N=191)	0.856	0.857	0.845	0.846	0.755	0.76	0.871	0.875	0.839	0.845	—	—	0.765	0.776	0.803	0.815
**Facility**																
Hospital (N=371)	0.811	0.812	0.831	0.833	0.813	0.816	0.905	0.908	0.875	0.876	0.920	0.927	0.775	0.786	0.838	0.843
Primary Care (N=540)	0.839	0.840	0.830	0.830	0.768	0.770	0.885	0.886	0.865	0.866	0.907	0.911	0.791	0.800	0.849	0.854
**Patient Status**																
Non-Patient (N=371)	0.784	0.786	0.800	0.801	0.716	0.720	0.890	0.892	0.857	0.858	0.929	0.932	0.782	0.794	0.757	0.774
Patient (N=479)	0.842	0.843	0.843	0.844	0.819	0.821	0.896	0.898	0.882	0.883	0.874	0.882	0.782	0.791	0.875	0.879

Note: α = Cronbach's alpha; ω_t = McDonald's omega; Alc = alcohol; Cann = cannabis.
[a]For certain strata, reliability measures for the ASSIST-Cannabis scale could not be computed due to no non-zero responses.

Table S2. Sensitivity and specificity of scales at different cutoff scores compared to corresponding MINI diagnoses.

	**PHQ-9**		**GAD-7**				**SSS-8**		**AUDIT**		**ASSIST**			
	**Depression**		**Anxiety**		**Panic**		**Somatization**		**Alcohol**		**Alcohol**		**Cannabis**	
**Score**	**Sens.**	**Spec.**	**Sens.**	**Spec.**	**Sens.**	**Spec.**	**Sens.**	**Spec.**	**Sens.**	**Spec.**	**Sens.**	**Spec.**	**Sens.**	**Spec.**
≥ 1	0.940	0.237	0.954	0.251	1.000	0.245	1.000	0.182	0.930	0.560	--	--	--	--
≥ 2	0.886	0.416	0.923	0.405	0.939	0.394	1.000	0.311	0.896	0.643	0.870	0.699	0.682	0.985
≥ 3	0.809	0.573	0.908	0.536	0.909	0.519	1.000	0.430	0.852	0.729	0.817	0.785	0.591	0.985
≥ 4	0.691	0.703	0.846	0.629	0.879	0.613	0.846	0.535	0.817	0.779	0.765	0.841	--	--
≥ 5	0.591	0.785	0.785	0.736	0.758	0.716	0.846	0.625	0.809	0.828	0.757	0.854	0.591	0.990
≥ 6	0.534	0.822	0.692	0.797	0.727	0.780	0.846	0.703	0.757	0.877	0.722	0.877	--	--
≥ 7	0.456	0.868	0.646	0.845	0.667	0.828	0.846	0.760	0.722	0.906	0.678	0.901	--	--
≥ 8	0.373	0.889	0.539	0.887	0.576	0.872	0.692	0.787	0.670	0.921	0.652	0.913	0.546	0.991
≥ 9	0.319	0.915	0.431	0.915	0.485	0.904	0.692	0.835	0.626	0.938	0.600	0.927	--	--
≥ 10	0.262	0.938	0.369	0.934	0.455	0.926	0.615	0.863	0.609	0.949	0.591	0.933	0.500	0.991
≥ 11	0.211	0.954	0.277	0.949	0.333	0.943	0.539	0.890	0.565	0.961	0.565	0.941	--	--
≥ 12	0.188	0.969	0.231	0.969	0.273	0.964	0.462	0.912	0.530	0.969	0.548	0.945	0.500	0.993
≥ 13	0.154	0.971	0.200	0.984	0.212	0.977	0.462	0.925	0.496	0.972	0.530	0.949	--	--
≥ 14	0.128	0.977	0.154	0.989	0.182	0.985	0.385	0.945	0.461	0.976	0.513	0.956	0.409	0.994
≥ 15	0.094	0.984	0.139	0.992	0.152	0.988	0.308	0.954	0.452	0.982	0.496	0.964	--	--
≥ 16	0.077	0.987	0.108	0.994	0.121	0.991	0.308	0.963	0.426	0.982	0.461	0.971	0.364	0.994
≥ 17	0.060	0.990	0.077	0.997	0.091	0.994	0.308	0.968	0.365	0.985	0.444	0.976	--	--
≥ 18	0.050	0.990	--	--	--	--	0.308	0.977	0.287	0.985	0.426	0.979	--	--
≥ 19	0.040	0.994	0.046	0.998	0.030	0.995	--	--	0.244	0.990	0.409	0.981	0.318	0.994
≥ 20	0.030	0.994	0.031	0.999	0.000	0.997	0.231	0.982	0.209	0.991	0.391	0.984	--	--
≥ 21	0.020	0.994	0.015	1.000	0.000	0.999	0.231	0.988	0.165	0.991	0.357	0.986	0.273	0.996
≥ 22	0.003	0.995	--	--	--	--	0.231	0.991	0.165	0.995	0.304	0.987	--	--
≥ 24	--	--	--	--	--	--	0.077	0.992	0.122	0.996	0.278	0.987	0.182	0.996
≥ 24	0.003	0.998	--	--	--	--	0.077	0.994	0.096	0.996	0.244	0.987	--	--
≥ 25	0.000	0.998	--	--	--	--	--	--	0.078	0.998	0.200	0.990	0.136	0.996
≥ 26	--	--	--	--	--	--	0.077	0.996	0.070	0.998	0.183	0.991	0.091	0.996
≥ 27	--	--	--	--	--	--	0.077	0.997	0.061	0.998	0.174	0.994	0.046	0.996
≥ 28	--	--	--	--	--	--	0.000	0.997	0.052	0.998	0.157	0.994	--	--
≥ 29	--	--	--	--	--	--	0.000	0.998	0.044	0.999	0.148	0.995	0.046	0.998
≥ 30	--	--	--	--	--	--	--	--	0.026	1.000	0.130	0.996	0.046	0.999
≥ 31	--	--	--	--	--	--	--	--	--	--	0.104	0.999	--	--
≥ 32	--	--	--	--	--	--	--	--	--	--	0.078	0.999	0.000	0.999
≥ 33	--	--	--	--	--	--	--	--	0.017	1.000	0.061	1.000	--	--
≥ 34	--	--	--	--	--	--	--	--	--	--	0.052	1.000	--	--
≥ 35	--	--	--	--	--	--	--	--	0.009	1.000	--	--	--	--
≥ 36	--	--	--	--	--	--	--	--	--	--	0.026	1.000	--	--
≥ 38	--	--	--	--	--	--	--	--	--	--	0.017	1.000	--	--

Note. Sens. = sensitivity; Spec. = specificity.

Table S3. Sensitivity and specificity of CSSR at different cutoff categories compared to moderate to high MINI suicide risk.

Score	CSSR Moderate-High Suicide Risk	
	Sens.	Spec.
≥ Low Risk	0.872	0.879
≥ Moderate Risk	0.535	0.960
≥ High Risk	0.417	0.984

Note. Sens. = sensitivity; Spec. = specificity.

Table S4. Performance of scales by gender, age, education level, primary language, facility-type, and patient status.

	PHQ-9	GAD-7		SSS-8	Audit	ASSIST		CSSR
	Depression	Anxiety	Panic	Somatization	Alcohol	Alcohol	Cannabis	Moderate-High Suicide Risk
	AUC (95% CI)	AUC (95% CI)	AUC (95% CI)	AUC (95% CI)	AUC (95%CI)	AUC (95%CI)	AUC (95% CI)	AUC (95% CI)
**Overall** (N=911)	0.754 (0.720, 0.787)	0.817 (0.762, 0.872)	0.828 (0.762, 0.895)	0.845 (0.747, 0.943)	0.883 (0.846, 0.919)	0.863 (0.823, 0.903)	0.834 (0.734, 0.934)	0.895 (0.856, 0.933)
**Gender**								
Female (N=570)	0.747 (0.705, 0.790)	0.818 (0.746, 0.889)	0.846 (0.774, 0.918)	0.854 (0.752, 0.957)	0.869 (0.806, 0.933)	0.821 (0.746, 0.896)	0.749 (0.464, 1.0)	0.925 (0.888, 0.963)
Male (N=341)	0.768 (0.714, 0.822)	0.816 (0.730, 0.903)	0.804 (0.678, 0.930)	0.793 (0.503, 1.00)	0.869 (0.819, 0.919)	0.867 (0.817, 0.917)	0.844 (0.737, 0.951)	0.832 (0.746, 0.917)
**Age**								
18-25 (N=324)	0.764 (0.710, 0.818)	0.759 (0.656, 0.863)	0.851 (0.771, 0.931)	0.920 (0.764, 1.00)	0.895 (0.834, 0.957)	0.881 (0.816, 0.947)	0.904 (0.794, 1)	0.908 (0.853, 0.963)
> 25 (N=587)	0.746 (0.703, 0.788)	0.850 (0.788, 0.913)	0.814 (0.721, 0.907)	0.826 (0.708, 0.944)	0.875 (0.829, 0.921)	0.853 (0.803, 0.903)	0.746 (0.582, 0.911)	0.883 (0.828, 0.938)
**Education**								
< Primary (N=183)	0.745 (0.671, 0.819)	0.669 (0.507, 0.830)	0.729 (0.530, 0.928)	0.924 (0.844, 1.00)	0.862 (0.777, 0.947)	0.899 (0.838, 0.959)	0.988 (0.973, 1)	0.901 (0.819, 0.983)
< Secondary (N=483)	0.746 (0.700, 0.793)	0.825 (0.762, 0.889)	0.821 (0.726, 0.916)	0.801 (0.590, 1.00)	0.914 (0.877, 0.951)	0.863 (0.808, 0.918)	0.801 (0.662, 0.939)	0.883 (0.826, 0.940)
≥ Secondary (N=244)	0.773 (0.706, 0.840)	0.936 (0.897, 0.976)	0.888 (0.802, 0.974)	0.871 (0.795, 0.948)	0.815 (0.700, 0.929)	0.827 (0.725, 0.929)	0.664 (0.335, 0.993)	0.913 (0.844, 0.981)
**Language**								
Portuguese only (N=517)	0.776 (0.734, 0.817)	0.830 (0.764, 0.896)	0.839 (0.751, 0.928)	0.816 (0.696, 0.937)	0.876 (0.825, 0.926)	0.880 (0.832, 0.929)	0.852 (0.746, 0.959)	0.900 (0.851, 0.949)
Portuguese + other (N=202)	0.765 (0.691, 0.839)	0.834 (0.744, 0.923)	0.909 (0.791, 1.00)	0.962 (0.919, 1.00)	0.902 (0.835, 0.968)	0.861 (0.774, 0.949)	0.993 (0.981, 1)	0.873 (0.760, 0.986)
Other only (N=191)	0.675 (0.590, 0.760)	0.665 (0.375, 0.954)	0.718 (0.585, 0.852)	0.505 (NA, NA)	0.883 (0.802, 0.963)	0.808 (0.670, 0.916)	0.497 (0.492, 0.503)	0.897 (0.821, 0.973)
**Facility**								
Hospital (N=371)	0.767 (0.718, 0.817)	0.821 (0.740, 0.902)	0.822 (0.731, 0.912)	0.849 (0.739, 0.959)	0.886 (0.823, 0.950)	0.878 (0.812, 0.944)	0.910 (0.747, 1)	0.887 (0.827, 0.948)
Primary Care (N=540)	0.738 (0.692, 0.784)	0.811 (0.741, 0.881)	0.829 (0.726, 0.931)	0.779 (0.504, 1.00)	0.878 (0.832, 0.923)	0.854 (0.804, 0.904)	0.806 (0.682, 0.929)	0.902 (0.852, 0.951)
**Patient Status**								
Non-Patient (N=371)	0.745 (0.695, 0.795)	0.833 (0.762, 0.904)	0.799 (0.659, 0.939)	0.844 (0.678, 1.00)	0.936 (0.900, 0.973)	0.902 (0.850, 0.955)	0.842 (0.692, 0.992)	0.900 (0.840, 0.961)
Patient (N=479)	0.769 (0.725, 0.813)	0.807 (0.725, 0.890)	0.835 (0.758, 0.910)	0.842 (0.711, 0.974)	0.844 (0.788, 0.900)	0.835 (0.778, 0.891)	0.828 (0.689, 0.967)	0.890 (0.839, 0.941)

Note. AUC = area under the ROC curve.

Table S4 cont.

	PSQ[a] Psychosis		PSQ[a] Mania	
	Sensitivity	Specificity	Sensitivity	Specificity
**Overall** (N=911)	0.579 (0.519, 0.643)	0.791 (0.760, 0.821)	0.729 (0.629, 0.829)	0.731 (0.702, 0.761)
**Gender**				
Female (N=570)	0.587 (0.500, 0.667)	0.801 (0.764, 0.836)	0.762 (0.619, 0.881)	0.744 (0.706, 0.780)
Male (N=341)	0.567 (0.464, 0.670)	0.775 (0.721, 0.824)	0.679 (0.500, 0.821)	0.709 (0.658, 0.760)
**Age**				
18-25 (N=324)	0.604 (0.505, 0.703)	0.794 (0.740, 0.848)	0.727 (0.576, 0.879)	0.715 (0.663, 0.766)
> 25 (N=587)	0.560 (0.478, 0.642)	0.790 (0.753, 0.828)	0.730 (0.595, 0.865)	0.740 (0.702, 0.775)
**Education**				
< Primary (N=183)	0.532 (0.383, 0.681)	0.802 (0.735, 0.868)	0.765 (0.529, 0.941)	0.765 (0.699, 0.825)
< Secondary (N=483)	0.654 (0.574, 0.735)	0.772 (0.726, 0.818)	0.702 (0.575, 0.830)	0.690 (0.645, 0.734)
≥ Secondary (N=244)	0.423 (0.289, 0.558)	0.823 (0.766, 0.875)	0.833 (0.500, 1.00)	0.786 (0.731, 0.836)
**Language**				
Portuguese only (N=517)	0.575 (0.500, 0.651)	0.757 (0.712, 0.798)	0.796 (0.682, 0.909)	0.706 (0.664, 0.744)
Portuguese + other (N=202)	0.561 (0.415, 0.707)	0.857 (0.801, 0.907)	0.500 (0.250, 0.750)	0.796 (0.737, 0.850)
Other only (N=191)	0.604 (0.458, 0.750)	0.804 (0.741, 0.867)	0.800 (0.500, 1.00)	0.729 (0.663, 0.790)
**Facility**				
Hospital (N=371)	0.520 (0.418, 0.622)	0.777 (0.725, 0.828)	0.704 (0.519, 0.852)	0.730 (0.686, 0.776)
Primary Care (N=540)	0.620 (0.533, 0.693)	0.802 (0.762, 0.839)	0.744 (0.605, 0.861)	0.732 (0.694, 0.771)
**Patient Status**				
Non-Patient (N=371)	0.523 (0.432, 0.613)	0.847 (0.810, 0.888)	0.839 (0.710, 0.968)	0.798 (0.758, 0.838)
Patient (N=479)	0.629 (0.548, 0.710)	0.741 (0.693, 0.786)	0.641 (0.487, 0.770)	0.671 (0.627, 0.711)

[a]Sensitivity and specificity were calculated instead of AUC because 0 and 1 are only possible scores on the PSQ.

Figure S1. Scree plot of exploratory factor analysis of all scales, excluding the ASSIST-Cannabis.

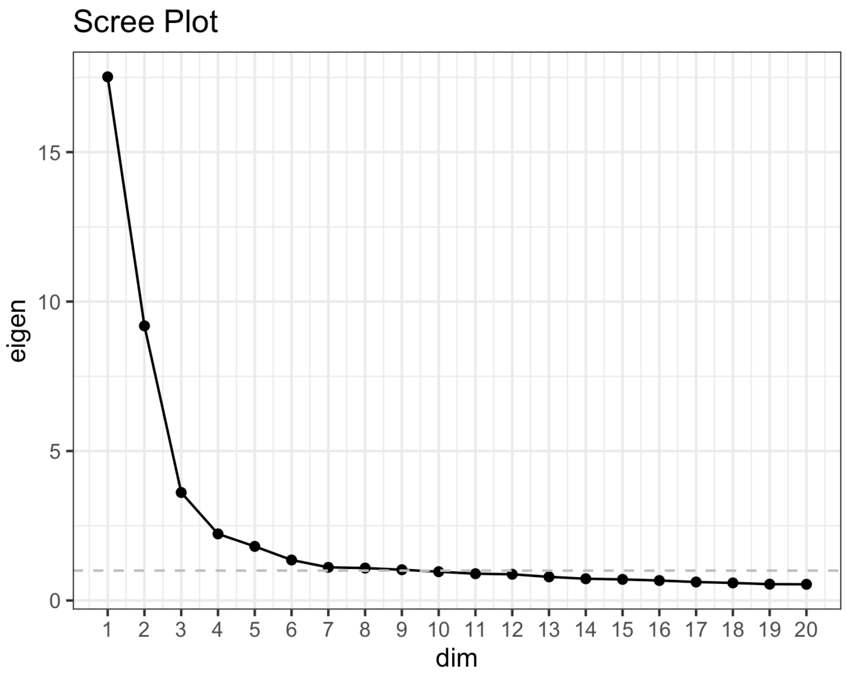

Table S5. Item loadings for 3-factor model of scales.

Item	Factor 1	Factor 2	Factor 3	Scale
PHQ1	0.562			PHQ
PHQ2	0.605		0.166	PHQ
PHQ3	0.746		-0.124	PHQ
PHQ4	0.817		-0.173	PHQ
PHQ5	0.631			PHQ
PHQ6	0.557		0.306	PHQ
PHQ7	0.638			PHQ
PHQ8	0.701			PHQ
PHQ9	0.485		0.495	PHQ
GAD1	0.652			GAD
GAD2	0.64			GAD
GAD3	0.582			GAD
GAD4	0.704			GAD
GAD5	0.665	0.116		GAD
GAD6	0.642			GAD
GAD7	0.591	0.129		GAD
SSS1	0.493			SSS
SSS2	0.557			SSS
SSS3	0.603	-0.106		SSS
SSS4	0.565			SSS
SSS5	0.623			SSS
SSS6	0.655			SSS
SSS7	0.774			SSS
SSS8	0.76			SSS
AUDIT1	-0.278	1.011		AUDIT
AUDIT2	-0.265	0.854		AUDIT
AUDIT3	-0.258	0.939		AUDIT
AUDIT4		0.848		AUDIT
AUDIT5		0.88	0.142	AUDIT
AUDIT6		0.85		AUDIT
AUDIT7		0.827		AUDIT
AUDIT8		0.823	0.159	AUDIT
AUDIT9		0.646		AUDIT
AUDIT10		0.844	-0.167	AUDIT
ASTALC1	-0.157	0.96	-0.166	ASSISTALC
ASTALC2		0.881		ASSISTALC
ASTALC3	0.12	0.805		ASSISTALC
ASTALC4	0.113	0.87		ASSISTALC
ASTALC5		0.905	-0.21	ASSISTALC
ASTALC6		0.891	-0.183	ASSISTALC
CSSRS1	0.232		0.729	CSSRS
CSSRS2			0.96	CSSRS
CSSRS3			0.924	CSSRS
CSSRS4			0.878	CSSRS
CSSRS5			0.929	CSSRS
CSSRS6			0.907	CSSRS
CSSRS7	0.219		0.886	CSSRS
PSQ1			0.265	PSQ
PSQ2	0.507			PSQ
PSQ3	0.392	0.149		PSQ
PSQ4	0.474			PSQ
PSQ5	0.468			PSQ

-0.25 0.00 0.25 0.50 0.75 1.00

Table S6. Item loadings for 4-factor model of scales.

Scale	Item	Factor 1	Factor 2	Factor 3	Factor 4
PHQ	PHQ1	0.476			0.177
PHQ	PHQ2	0.51		0.158	0.187
PHQ	PHQ3	0.649		-0.153	0.214
PHQ	PHQ4	0.796		-0.157	
PHQ	PHQ5	0.56			0.143
PHQ	PHQ6	0.427		0.293	0.255
PHQ	PHQ7	0.579			
PHQ	PHQ8	0.58			0.253
PHQ	PHQ9	0.354		0.492	0.236
GAD	GAD1	0.646			
GAD	GAD2	0.63			
GAD	GAD3	0.541			
GAD	GAD4	0.71			
GAD	GAD5	0.614	0.122	0.11	
GAD	GAD6	0.589	0.089		
GAD	GAD7	0.524	0.13		0.146
SSS	SSS1	0.494			
SSS	SSS2	0.637			-0.213
SSS	SSS3	0.71			-0.278
SSS	SSS4	0.671			-0.268
SSS	SSS5	0.698			-0.213
SSS	SSS6	0.725			-0.198
SSS	SSS7	0.839			-0.179
SSS	SSS8	0.693			0.142
AUDIT	AUDIT1		0.993		-0.346
AUDIT	AUDIT2		0.839	0.101	-0.26
AUDIT	AUDIT3		0.923		-0.268
AUDIT	AUDIT4		0.835		
AUDIT	AUDIT5		0.854	0.154	0.147
AUDIT	AUDIT6		0.842		
AUDIT	AUDIT7		0.816		
AUDIT	AUDIT8		0.806	0.174	
AUDIT	AUDIT9		0.635		
AUDIT	AUDIT10		0.825	-0.136	0.152
ASSISTALC	ASTALC1		0.95	-0.152	-0.216
ASSISTALC	ASTALC2	0.118	0.876		-0.116
ASSISTALC	ASTALC3	0.139	0.792		
ASSISTALC	ASTALC4		0.85		0.153
ASSISTALC	ASTALC5		0.888	-0.181	0.15
ASSISTALC	ASTALC6		0.877	-0.171	
CSSRS	CSSRS1			0.737	0.154
CSSRS	CSSRS2			0.97	
CSSRS	CSSRS3			0.929	
CSSRS	CSSRS4			0.886	
CSSRS	CSSRS5			0.937	
CSSRS	CSSRS6	-0.148		0.904	
CSSRS	CSSRS7			0.856	0.395
PSQ	PSQ1			0.25	0.313
PSQ	PSQ2				0.62
PSQ	PSQ3		0.128		0.428
PSQ	PSQ4				0.645
PSQ	PSQ5				0.639

0.0 0.5

Table S7. Item loadings for 5-factor model of scales.

Scale	Item	Factor 1	Factor 2	Factor 3	Factor 4	Factor 5
PHQ	PHQ1	0.491				0.166
PHQ	PHQ2	0.556		0.167		0.137
PHQ	PHQ3	0.636		-0.15		0.239
PHQ	PHQ4	0.656		-0.146	0.291	0.19
PHQ	PHQ5	0.463			0.162	0.26
PHQ	PHQ6	0.42		0.299		0.272
PHQ	PHQ7	0.52			0.126	0.182
PHQ	PHQ8	0.533				0.313
PHQ	PHQ9	0.337		0.497		0.268
GAD	GAD1	0.726				
GAD	GAD2	0.735				
GAD	GAD3	0.658			-0.141	
GAD	GAD4	0.808				
GAD	GAD5	0.681	0.128			
GAD	GAD6	0.674				
GAD	GAD7	0.541	0.133			0.126
SSS	SSS1	0.4			0.199	
SSS	SSS2	0.458			0.399	
SSS	SSS3	0.484			0.507	
SSS	SSS4	0.543			0.358	-0.131
SSS	SSS5	0.476			0.478	
SSS	SSS6	0.53			0.415	
SSS	SSS7	0.662			0.406	
SSS	SSS8	0.645			0.121	0.203
AUDIT	AUDIT1		1.005			-0.386
AUDIT	AUDIT2		0.848	0.109		-0.302
AUDIT	AUDIT3		0.934			-0.302
AUDIT	AUDIT4		0.84			
AUDIT	AUDIT5		0.855	0.155		0.136
AUDIT	AUDIT6		0.85		0.164	
AUDIT	AUDIT7		0.823			
AUDIT	AUDIT8		0.811	0.179		
AUDIT	AUDIT9		0.638			
AUDIT	AUDIT10		0.827		-0.174	
ASSISTALC	ASTALC1		0.961	-0.153		-0.261
ASSISTALC	ASTALC2	0.127	0.886			-0.143
ASSISTALC	ASTALC3		0.798			
ASSISTALC	ASTALC4		0.853			0.178
ASSISTALC	ASTALC5		0.891	-0.165	-0.144	0.103
ASSISTALC	ASTALC6		0.883	-0.168		
CSSRS	CSSRS1			0.739		0.21
CSSRS	CSSRS2			0.974		
CSSRS	CSSRS3			0.93		
CSSRS	CSSRS4			0.889		
CSSRS	CSSRS5			0.94		
CSSRS	CSSRS6			0.888	-0.24	
CSSRS	CSSRS7	0.354		0.807	-0.475	
PSQ	PSQ1			0.281		0.562
PSQ	PSQ2					0.661
PSQ	PSQ3					0.477
PSQ	PSQ4					0.729
PSQ	PSQ5					0.77

Color scale: 0.0 0.5 1.0

Table S8. Item loadings for 6-factor model of scales.

Scale	Item	Factor 1	Factor 2	Factor 3	Factor 4	Factor 5	Factor 6
PHQ	PHQ1	0.349			0.217		0.197
PHQ	PHQ2	0.354		0.148	0.206		0.302
PHQ	PHQ3	0.456				0.56	
PHQ	PHQ4	0.703		-0.18	0.21		
PHQ	PHQ5	0.456			0.273		
PHQ	PHQ6	0.289		0.284	0.324		0.169
PHQ	PHQ7	0.473			0.198	0.104	
PHQ	PHQ8	0.437			0.359		
PHQ	PHQ9	0.224		0.49	0.314		0.149
GAD	GAD1	0.53					0.394
GAD	GAD2	0.481					0.445
GAD	GAD3	0.386					0.432
GAD	GAD4	0.576				0.145	0.407
GAD	GAD5	0.46	0.109				0.369
GAD	GAD6	0.439					0.409
GAD	GAD7	0.404	0.113		0.214	-0.102	0.272
SSS	SSS1	0.452					
SSS	SSS2	0.645					-0.138
SSS	SSS3	0.739					-0.2
SSS	SSS4	0.682			-0.123		
SSS	SSS5	0.721					-0.195
SSS	SSS6	0.712					
SSS	SSS7	0.799					
SSS	SSS8	0.555				0.526	
AUDIT	AUDIT1		1.004		-0.412		
AUDIT	AUDIT2		0.841	0.12	-0.305	-0.112	
AUDIT	AUDIT3		0.93		-0.297	-0.14	
AUDIT	AUDIT4		0.849				
AUDIT	AUDIT5		0.866	0.168		0.14	
AUDIT	AUDIT6		0.858				-0.197
AUDIT	AUDIT7		0.823				
AUDIT	AUDIT8		0.815	0.189			
AUDIT	AUDIT9		0.633				
AUDIT	AUDIT10		0.815		0.137	-0.207	0.127
ASSISTALC	ASTALC1		0.965	-0.152	-0.279		
ASSISTALC	ASTALC2		0.888		-0.149		
ASSISTALC	ASTALC3		0.806			0.236	
ASSISTALC	ASTALC4		0.86		0.142	0.157	
ASSISTALC	ASTALC5		0.884	-0.165	0.16	-0.216	
ASSISTALC	ASTALC6		0.887	-0.165			
CSSRS	CSSRS1			0.738	0.23		
CSSRS	CSSRS2			0.976			
CSSRS	CSSRS3			0.929			
CSSRS	CSSRS4			0.887			
CSSRS	CSSRS5			0.947			
CSSRS	CSSRS6			0.878		0.353	
CSSRS	CSSRS7			0.747		0.58	
PSQ	PSQ1			0.29	0.486		-0.588
PSQ	PSQ2				0.73		
PSQ	PSQ3		0.126		0.518		
PSQ	PSQ4				0.789		
PSQ	PSQ5				0.785		-0.208

-0.5 0.0 0.5 1.0

Table S9. Item loadings for 7-factor model of scales.

Scale	Item	Factor 1	Factor 2	Factor 3	Factor 4	Factor 5	Factor 6	Factor 7
PHQ	PHQ1	0.312						0.409
PHQ	PHQ2	0.332		0.164		0.176	0.256	0.212
PHQ	PHQ3	0.473			0.59			
PHQ	PHQ4	0.694		-0.162				0.175
PHQ	PHQ5	0.44				0.199		0.245
PHQ	PHQ6	0.268		0.296		0.299		0.191
PHQ	PHQ7	0.454						0.306
PHQ	PHQ8	0.401				0.264		0.436
PHQ	PHQ9	0.2		0.511		0.294		0.199
GAD	GAD1	0.522					0.417	
GAD	GAD2	0.466					0.438	
GAD	GAD3	0.374					0.43	
GAD	GAD4	0.57			0.177		0.425	
GAD	GAD5	0.452					0.384	
GAD	GAD6	0.431				0.133	0.428	
GAD	GAD7	0.398	0.093			0.237	0.288	
SSS	SSS1	0.45						
SSS	SSS2	0.651						
SSS	SSS3	0.744					-0.161	
SSS	SSS4	0.683				-0.151		
SSS	SSS5	0.732					-0.141	
SSS	SSS6	0.723						
SSS	SSS7	0.797						
SSS	SSS8	0.583			0.577			
AUDIT	AUDIT1		1.005			-0.393		
AUDIT	AUDIT2		0.825			-0.271		-0.166
AUDIT	AUDIT3		0.919			-0.268		-0.158
AUDIT	AUDIT4		0.847					
AUDIT	AUDIT5		0.842	0.12	0.22	0.136		
AUDIT	AUDIT6		0.855				-0.168	
AUDIT	AUDIT7		0.808					
AUDIT	AUDIT8		0.79	0.147	0.155			
AUDIT	AUDIT9		0.623					
AUDIT	AUDIT10		0.8			0.17	0.149	
ASSISTALC	ASTALC1		0.992	-0.101	-0.101	-0.305		0.231
ASSISTALC	ASTALC2		0.902			-0.161		0.21
ASSISTALC	ASTALC3		0.82					0.323
ASSISTALC	ASTALC4		0.865			0.135	-0.145	0.231
ASSISTALC	ASTALC5		0.879	-0.143	-0.207	0.179		
ASSISTALC	ASTALC6		0.891					
CSSRS	CSSRS1			0.737		0.269		
CSSRS	CSSRS2			0.982				
CSSRS	CSSRS3			0.929				
CSSRS	CSSRS4			0.901				
CSSRS	CSSRS5			0.941				
CSSRS	CSSRS6			0.876				
CSSRS	CSSRS7			0.779				0.663
PSQ	PSQ1			0.292		0.46	-0.598	
PSQ	PSQ2					0.73		
PSQ	PSQ3					0.54		
PSQ	PSQ4					0.85		
PSQ	PSQ5					0.768	-0.221	

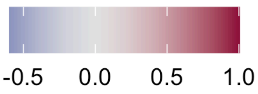

Supplement: Supplementary material [file NIHMS2020651-supplement-Supplementary_material.pdf]
